# Spontaneous Rupture of a Hepatic Adenoma: Diagnostic Nuances and the Necessity of Followup

**DOI:** 10.7759/cureus.1975

**Published:** 2017-12-20

**Authors:** Preston F Ashby, Chelsea Alfafara, Albert Amini, Richard Amini

**Affiliations:** 1 Department of Surgery, Arizona College of Osteopathic Medicine; 2 College of Medicine, University of Arizona; 3 Arizona Premier Surgery, Chandler Regional Hospital; 4 Department of Emergency Medicine, University of Arizona

**Keywords:** hepatic hemangioma, focal nodular hyperplasia, hepatic adenoma

## Abstract

We present the case of a young female on oral contraceptives (OCs) who was diagnosed with focal nodular hyperplasia (FNH) and remained on oral contraceptives. Months later, the patient presented with acute abdominal pain and intratumoral hemorrhage in the liver. The patient was taken to the operating room (OR) and was diagnosed with a ruptured hepatic adenoma (HA). We review the key diagnostic features of FNH and HA, the different management guidelines including use of OCs, and potential surgical indications. HA compared to FNH has a significantly higher rate of sequelae despite being a benign lesion, thus providers must accurately distinguish between the two diagnoses to prevent potential morbidity and mortality.

## Introduction

After hepatic hemangioma, focal nodular hyperplasia (FNH) and hepatic adenoma (HA) are the second and third most common benign liver neoplasms, respectively. Both tumors are typically asymptomatic and are usually discovered incidentally on imaging. HA is strongly associated with exposure to estrogens, particularly estrogen-containing oral contraceptives (OCs) [1]. Overall, up to 30% of HA tumors are associated with rupture or hemorrhage, and this risk is highest in patients with hormone use and in patients with larger tumors (>5 cm) [2]. If bleeding is severe, emergent surgery must be performed, which carries a 5-10% mortality rate. This is significantly higher than elective tumor resections which have a mortality rate of 1%. Malignant transformation of HAs has been documented as high as 5% [3], whereas FNH has no known malignant potential and few, if any, complications.

## Case presentation

**Figure 1:**
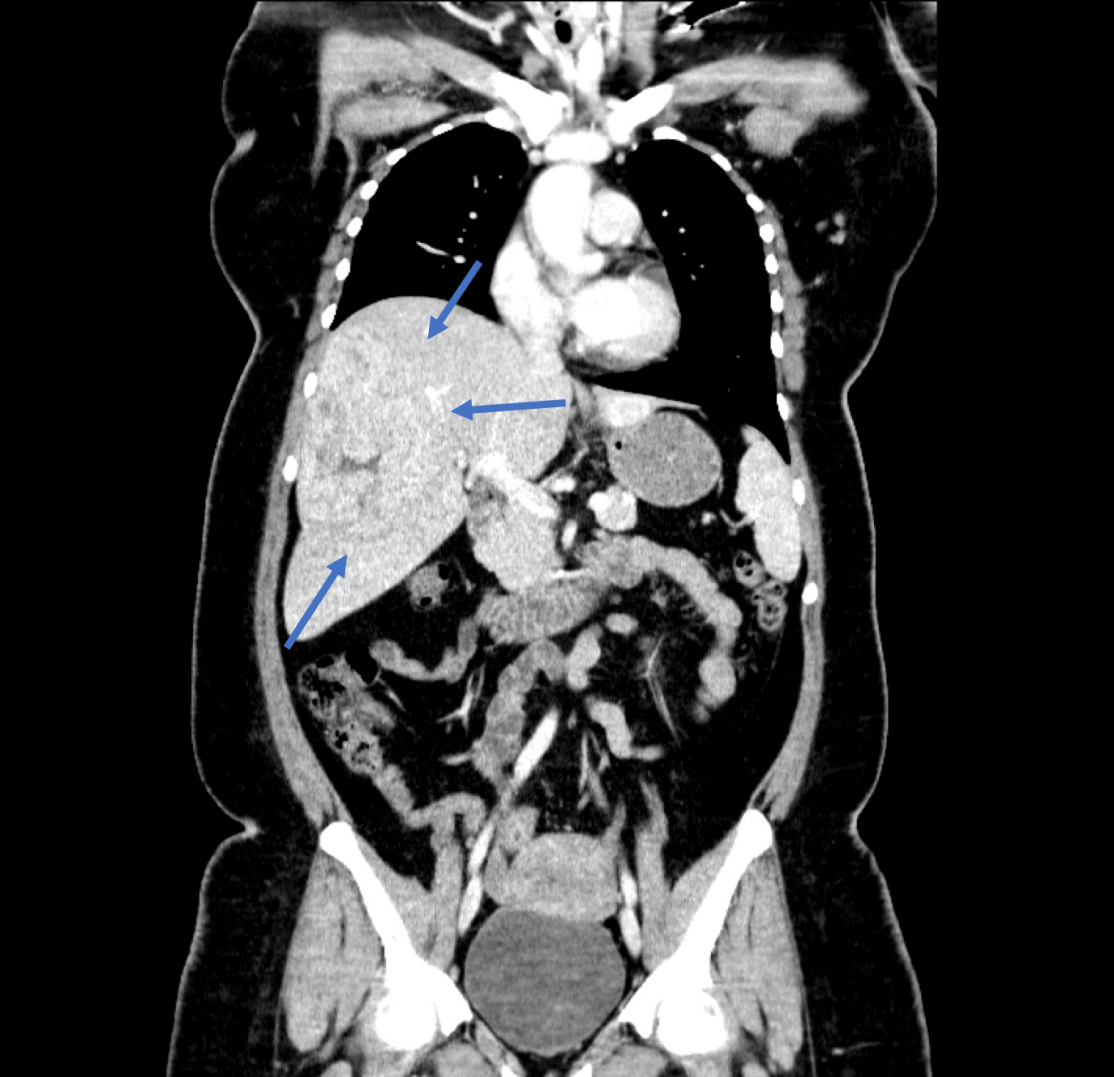
Computed tomography one year prior to presentation. Computed tomography demonstrates a 12.6 cm x 12.1 cm x 8.6 cm right liver mass with central stellate scar present, consistent with focal nodular hyperplasia (FNH). Followup with non-emergent outpatient magnetic resonance imaging (MRI) recommended.

A 31-year-old Caucasian female presented to the emergency department with acute right upper quadrant (RUQ) abdominal pain over the past day. The patient described her pain as constant, sharp, and burning, with radiation to the back. The patient also complained of nausea and non-bloody, non-bilious emesis. Her past medical history was significant for hypertension and a liver mass consistent with FNH that was incidentally noted on abdominal computed tomography (CT) over a year ago (Figure 1).

Although the patient was given instructions to follow up with her primary care physician (PCP) to review the liver mass findings and potentially receive outpatient magnetic resonance imaging (MRI), the patient did not follow up. The patient’s relevant medications were OC use for the past six years. On physical examination, there was hepatomegaly and significant tenderness to palpation over the RUQ. The patient did not exhibit rebound tenderness, guarding or Murphy's sign.

**Figure 2:**
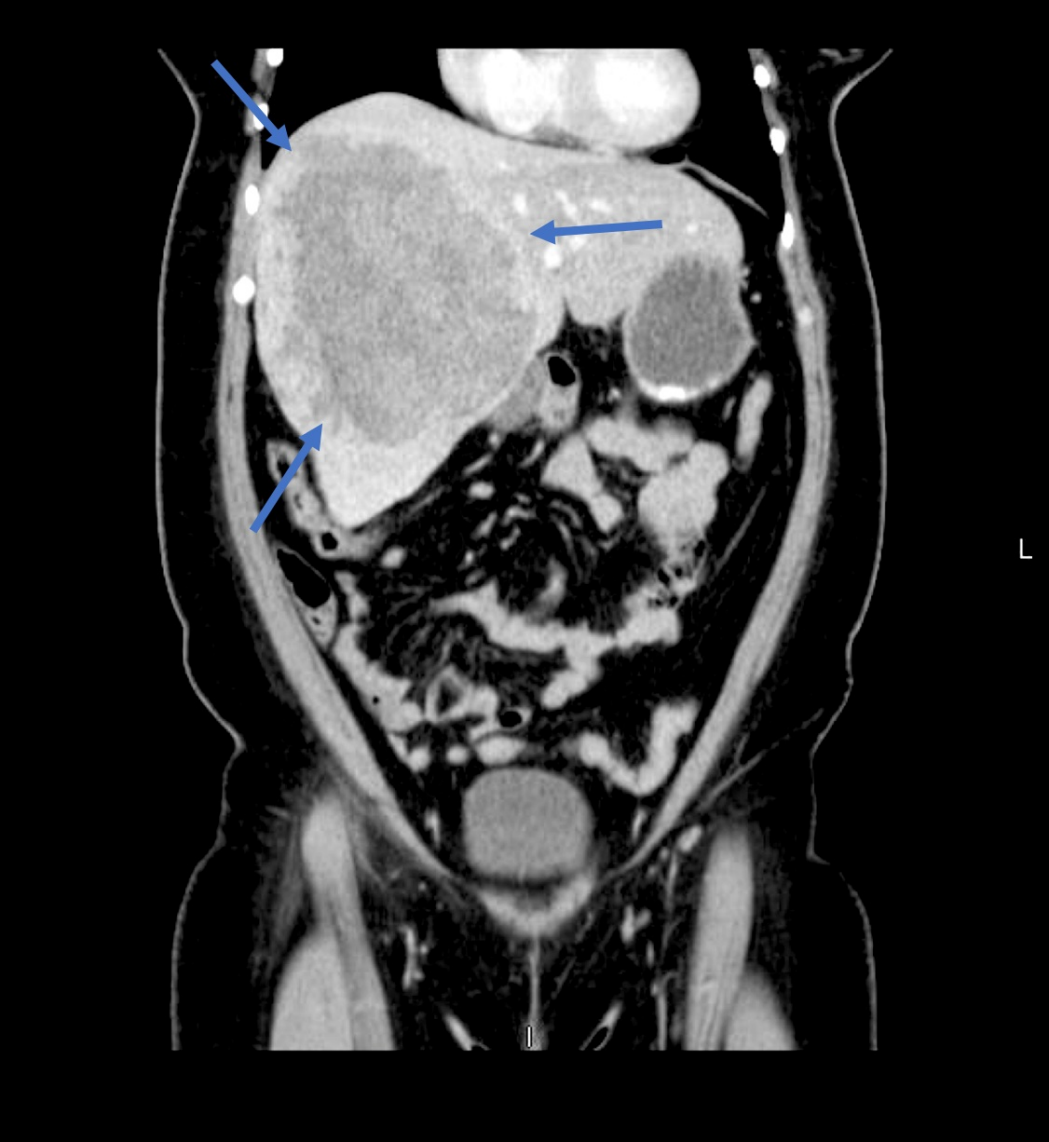
Computed tomography on the day of presentation. Computed tomography demonstrating a 16 cm x 13 cm x 13 cm right liver mass with central stellate scar consistent with focal nodular hyperplasia (FNH).

Initial work-up included a negative pregnancy test, elevated liver enzymes with an Alkaline phosphatase level of 116 IU/L, Aspartate Aminotransferase (AST) of 160 IU/L, Alanine Aminotransferase (ALT) of 281 IU/L, and total bilirubin of 0.5 mg/dL. White blood cell count (WBC) was slightly elevated at 12,000 per microliter and her hemoglobin level was 11.5 g/dL. The international normalized ratio (INR) was 1.1 and Creatinine was 0.77 mg/dL. CT scan of the abdomen with contrast demonstrated the same large heterogeneous mass in the right lobe of the liver (Figure 2) without acute pathology to explain the patient's pain. RUQ ultrasound demonstrated the same findings and there were no signs of acute cholecystitis.

**Figure 3:**
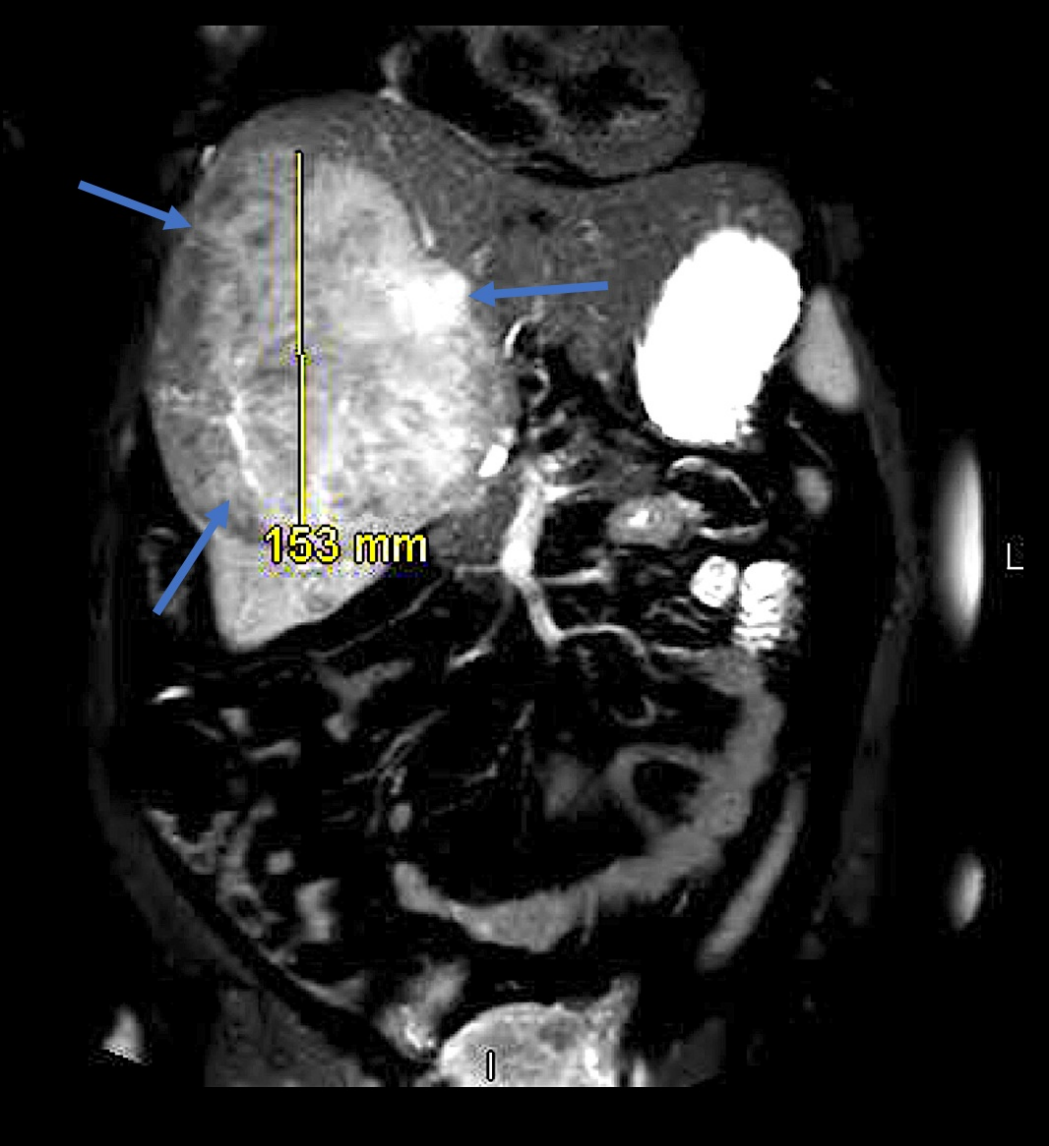
Magnetic resonance imaging of the abdomen. The magnetic resonance imaging (MRI) of the abdomen demonstrated a 15.3 cm x 12.5 cm x 14.1 cm heterogeneous mass straddling the right and left lobe of the liver. There was also peripheral enhancement consistent with hemorrhage.

The emergency physician admitted the patient for intractable pain and hematology/oncology was consulted as a result of the liver lesion. On the subsequent day, the patient's pain continued and repeat blood test demonstrated a drop in hemoglobin level to 8.1 g/dL. Concern for internal bleeding led physicians to order an MRI of the abdomen with and without contrast, which demonstrated a 15.3 cm x 12.5 cm x 14.1 cm heterogeneous mass straddling the right and left lobe of the liver. There was heterogeneous T1 and T2 hyperintensity throughout the entire mass, and mild peripheral enhancement consistent with hemorrhage. In addition, three smaller lesions with T1 hyperintensity, about 2 cm in size, were noted adjacent to the larger mass (Figure 3).

Interventional radiology performed a biopsy of the large liver mass. Histopathology results from the biopsy revealed hepatocellular neoplasm with extensive necrosis. CD34 stain showed increased vascularity. Due to the history of OC use and the characteristics of the liver mass found on imaging, the leading differentials were HA or a well differentiated hepatocellular carcinoma (HCC) or FNH.

**Figure 4:**
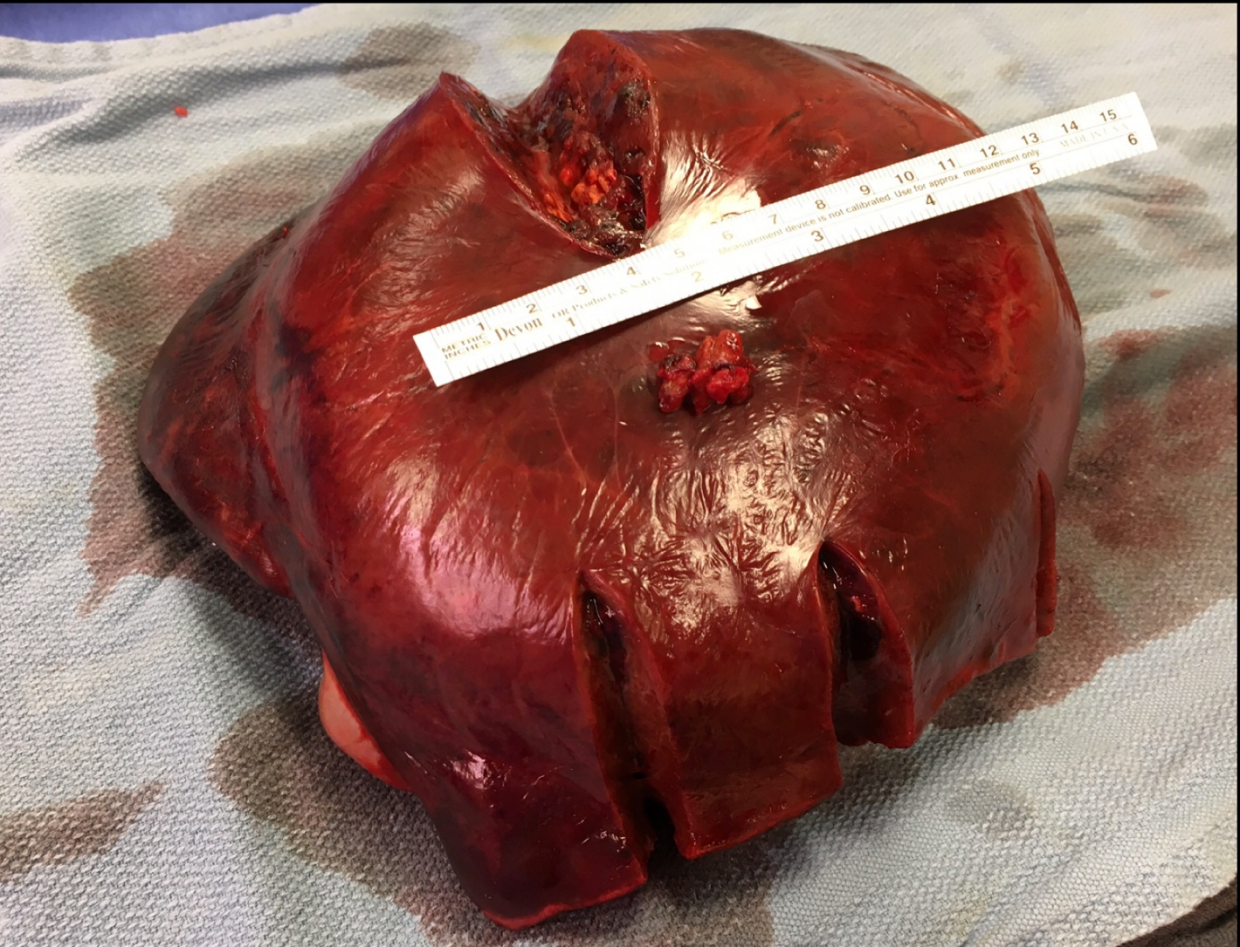
Postoperative image of the resected hepatic adenoma. The patient underwent a laparoscopic-assisted extended right hepatectomy including parts of segment 4 and 1.

The patient underwent a laparoscopic-assisted extended right hepatectomy including parts of segment 4 and 1 (Figure 4).

There were no intraoperative complications, she recovered well, and was discharged on postoperative day 4. Her postoperative labs demonstrated improved liver function enzymes and normal WBC. The histopathology of the surgical specimen was identified as a hepatic adenoma with surrounding hemorrhage and negative resection margins. Beta-catenin was negative which means the patient is at a lower risk for malignant transformation.

The patient returned to the surgery clinic for postoperative followup. Her repeat liver function tests decreased to an AST of 34 IU/L and an ALT of 87 IU/L. Her WBC normalized to 6,700 per microliter. She had no peri-operative or post-operative complications. She plans to schedule an appointment with the gynecologist for alternative contraceptive options.

## Discussion

FNH is a benign neoplasm that affects all ages and preferentially affects women over men (8-9:1). Currently, it is the second most common cause of benign hepatic tumors in adults behind hepatic hemangiomas [3]. Although the pathophysiology of FNH is not fully understood, a proposed mechanism is that the tumor arises from locally disturbed blood flow, which subsequently causes a hyperplastic, polyclonal response in normal hepatocytes as a result of either hypoxia or hyperperfusion [3]. Though the role of OCs in FNH is not clear, OCs may contribute to the growth of the tumor [4].

Histologically, FNH is described as a focal form of cirrhosis with a central fibrous scar, ductular proliferation, and malformed vessels. The central fibrous scar may manifest on CT scan or MRI as a central stellate scar, which is pathognomonic for FNH. Other imaging characteristics typically include a mass less than 5 cm in size [5], lobulated, but well demarcated, and homogeneity with slight hypo- or isoattenuation compared to the surrounding liver [5]. MRI will demonstrate hypo- or isoattenuation on T1 weighted images, but will show hyper- or isoattenuation on T2 weighted images [5]. MRI stands to be the most sensitive (70%) and specific (98-100%) diagnostic imaging technique. Furthermore, it is preferred over CT so as to avoid radiation in women of childbearing age [3]. Though CT scan with contrast is a reasonable alternative to MRI, there can be atypical characteristics that make the distinction between FNH and HA difficult [6]. Lastly, ultrasound is not a sufficient imaging modality to distinguish between FNH and HA; however, ultrasound with contrast has shown promise as an accurate and inexpensive imaging modality in the diagnosis of FNH [7]. When results of an abdominal MRI/CT are inconclusive, the patient may then be referred for a liver biopsy.

FNH is typically asymptomatic; the lesion is usually found incidentally, and only 12-13% of patients reporting abnormal serum liver tests. Rare symptoms include palpable abdominal mass (2-4%), hepatomegaly (<1%), and fever (<1%) [3]. When asymptomatic, FNH does not usually require surgical resection due to a very low risk of complications. The American College of Gastroenterology (ACG) recommends annual ultrasonography to monitor the lesion if the patient decides to continue OCs. In the absence of OCs and a firm diagnosis of FNH, the patient requires no further followup. Furthermore, if the patient is asymptomatic, surgery is not recommended [8].

Behind hepatic hemangioma and FNH, HA is the third most common cause of benign liver neoplasms and is found almost exclusively in women between the second to fifth decade of life. Since the 1970s, with the advent of OCs, the incidence of HA in young women has increased [9]. A case-control study conducted by Rooks, et al., estimates the risk of HA for long-term users of low-potency OCs correlates with an annual incidence of 3.4 per 100,000 [1]. Although the exact disease mechanism is largely unknown, HAs association with young women on oral contraceptives may suggest an estrogen-induced pathologic mechanism. Furthermore, there is convincing evidence between dose and duration of OCs with incidence of HA, adenoma size, and malignant transformation risk [1]. Other less-documented associations to HA are the use of anabolic androgenic steroids by bodybuilders and glycogen storage disease [3].

HAs are reported to be solitary lesions (70-80% of cases), which lack an obvious fibrous capsule on imaging. Histologically, these neoplasms are characterized by the presence of liver cell plates in the absence of bile ducts and fibrosis [3]. Aberrant vascular structures are extensive within the sinusoids supplied by the peripheral arterial system but lacking in supply by the portal venous system. Unlike FNH, hepatic adenomas do not feature bile ductules or other portal tract elements or fibrosis [3]. Imaging modalities used to diagnose HA are multiphase CT and MRI. Liver biopsy following inconclusive imaging studies remains controversial due to the increased risk of bleeding and hemorrhage [3].

Like those with FNH, a majority of patients with HA are often asymptomatic with up to 7% of cases reporting abnormal serum liver tests. Thirty to 40% of patients report abdominal discomfort and 2-4% have a palpable abdominal mass [3]. These patients, however, are more likely to present with acute abdominal pain after tumor rupture or hemorrhage. Up to a 30% incidence of spontaneous bleeding is reported and 5% progression to hepatocellular carcinoma in HA cases, and thus, surgical resection is the preferred choice of treatment. If a suspected HA is less than 5 cm in size, it can be monitored without surgery. However, followup imaging is recommended. Repeat CT or MRI at 6-12 month intervals is required and duration of followup depends on the stability of the HA over time [8]. Cessation of OCs, including progesterone only OCs, is recommended in all patients with HA [3, 9]. Alternative contraception is limited to copper intrauterine device, barrier methods, or more permanent methods such as tubal ligation, vasectomy, or hysterectomy.

**Figure 5:**
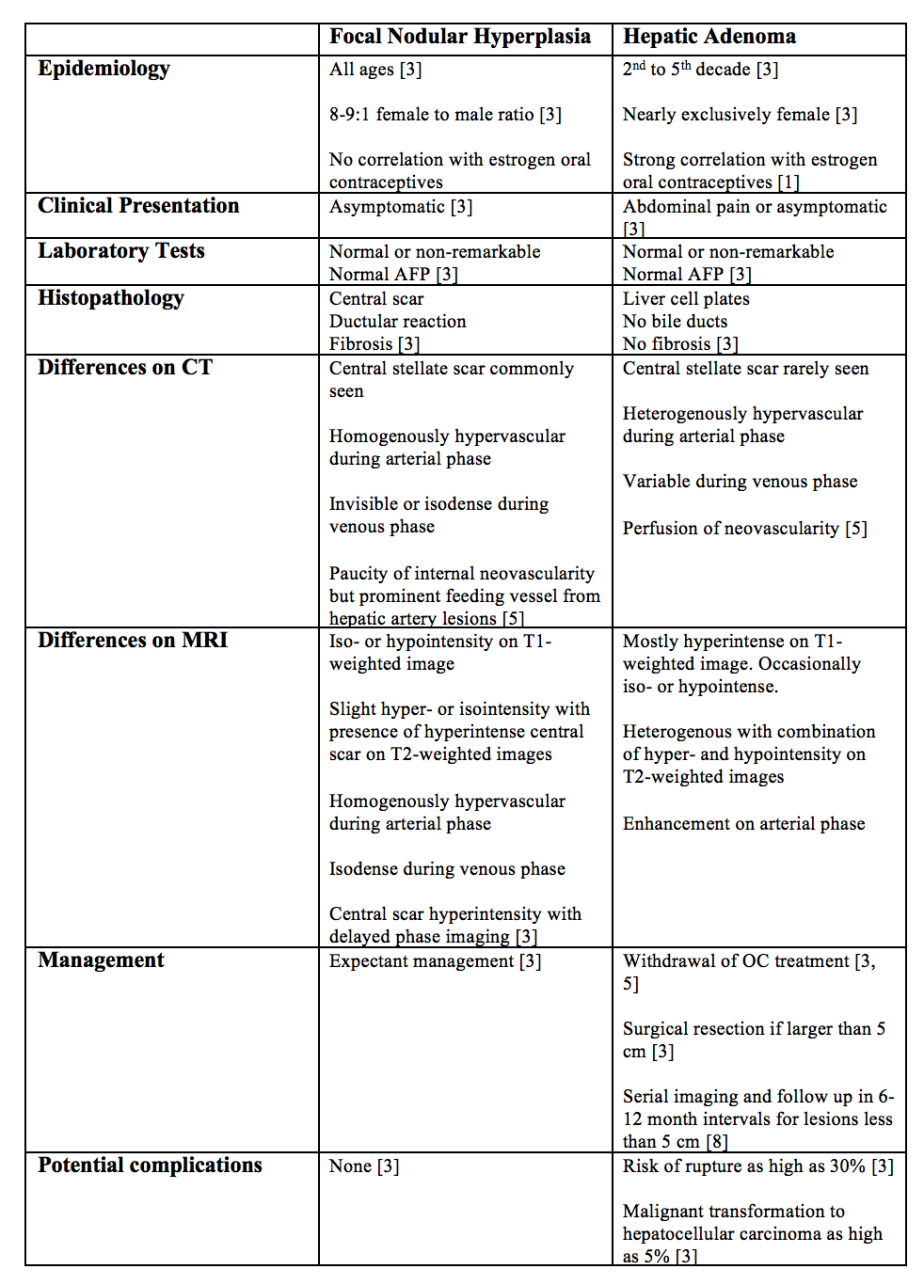
Differentiating focal nodular hyperplasia from hepatic adenoma.

Various epidemiologic, histologic, diagnostic, and clinical management differences exist between FNA and HA, and these differences are highlighted in Figure 5.

In our case, the patient was diagnosed with an FNH based on CT imaging. An MRI was recommended for followup, but the patient did not follow up and did not receive additional imaging. As discussed, atypical lesions may be difficult to diagnose on CT and thus require MRI. A diagnosis of HA could have led to earlier termination of her OCs. Although unlikely to have prevented surgery, due to the large size of the tumor, appropriate followup and imaging may have led to the reduction in the size of the liver resection and higher likelihood of complete laparoscopic resection.

## Conclusions

Although HA and FNH are common hepatic lesions with numerous similaries, clinicians should be aware of the diagnostic differences, preferred imaging modalities, and clinical management differences. Clinicians should have discussions with their patients with regard to the use of OCs, the necessity of close outpatient followup, choice of imaging modalities, and the potential need for surgical consultation.
